# Does predictability matter? Effects of cue predictability on neurocognitive mechanisms underlying prospective memory

**DOI:** 10.3389/fnhum.2015.00188

**Published:** 2015-04-13

**Authors:** Giorgia Cona, Giorgio Arcara, Vincenza Tarantino, Patrizia S. Bisiacchi

**Affiliations:** ^1^Department of General Psychology, University of PaduaPadua, Italy; ^2^Department of Neuroscience, University of PaduaPadua, Italy; ^3^Center for Cognitive Neuroscience, University of PaduaPadua, Italy

**Keywords:** prospective memory, strategic monitoring, ERPs, predictability, intention, dynamic multiprocess framework, AtoDI model, neural

## Abstract

Prospective memory (PM) represents the ability to successfully realize intentions when the appropriate moment or cue occurs. In this study, we used event-related potentials (ERPs) to explore the impact of cue predictability on the cognitive and neural mechanisms supporting PM. Participants performed an ongoing task and, simultaneously, had to remember to execute a pre-specified action when they encountered the PM cues. The occurrence of the PM cues was predictable (being signaled by a warning cue) for some participants and was completely unpredictable for others. In the predictable cue condition, the behavioral and ERP correlates of strategic monitoring were observed mainly in the ongoing trials wherein the PM cue was expected. In the unpredictable cue condition they were instead shown throughout the whole PM block. This pattern of results suggests that, in the predictable cue condition, participants engaged monitoring only when subjected to a context wherein the PM cue was expected, and disengaged monitoring when the PM cue was not expected. Conversely, participants in the unpredictable cue condition distributed their resources for strategic monitoring in more continuous manner. The findings of this study support the most recent views—the “Dynamic Multiprocess Framework” and the “Attention to Delayed Intention” (AtoDI) model—confirming that strategic monitoring is a flexible mechanism that is recruited mainly when a PM cue is expected and that may interact with bottom-up spontaneous processes.

## Introduction

Remembering to accomplish an intended action at the appropriate time or situation in the future is typically referred to as Prospective Memory (PM; Brandimonte et al., 1996). Remembering to take a medication after lunch, or returning a library book by the due date are everyday examples of what has been termed PM. Yet, the processes underlying prospective remembering are a matter of debate (Smith, 2003; Einstein and McDaniel, 2010). In particular, there is still a discussion on the role of strategic monitoring processes in maintaining delayed intentions and in identifying the appropriate situation in which the action has to be executed. In experimental contexts, the recruitment of strategic monitoring is indexed by the cost of adding a PM task to the ongoing activity and is labeled “PM interference effect” (e.g., Marsh et al., 2003; Smith, 2003; Hicks et al., 2005). Indeed, when strategic monitoring is engaged, it utilizes resources intended for the ongoing task, leading to a decline in performance, as indicated by the slowing of reaction times (RTs) and/or the decrease in accuracy. Guynn (2003) suggested a two-process model of strategic monitoring, which assumes that the PM interference effect is the result of a retrieval mode, mediating the maintenance of intention, and target checking, subserving the identification of the PM cue. Moreover, a third process—the readiness mode—has been hypothesized to be involved in PM task. It consists in being in a promptness state in order to execute the intention when the upcoming PM cue occurs (Cona et al., 2012a).

It is still unclear how such processes are engaged for the purpose of accomplishing PM intentions and whether they are always necessary. One group of theories proposes that strategic monitoring is always required to detect the occurrence of the PM cue in the environment (Smith, 2003; Smith and Bayen, 2004), whereas the Multiprocess Framework (e.g., Einstein and McDaniel, 2005; Scullin et al., 2010) states that delayed intentions can be retrieved either spontaneously or by recruiting top-down monitoring processes depending on a multitude of factors. Some of the factors that modulate the extent to which strategic monitoring is involved are the salience, valence, frequency, and focality of the PM cue (Einstein et al., 2005; McDaniel and Einstein, 2007; Czernochowski et al., 2012; Cona et al., 2014, 2015a).

Recently, an update of the Multiprocess Framework has been proposed: the “Dynamic Multiprocess Framework” (Scullin et al., 2013). According to this theory, strategic monitoring and spontaneous retrieval are not mutually exclusive but they might interplay dynamically to mediate performance on PM tasks. More specifically, strategic monitoring would be engaged when the occurrence of the PM cues is expected and would be disengaged when its occurrence is not expected. In the latter condition, individuals may however rely on a probabilistic spontaneous retrieval process in order to accomplish PM intentions. The Dynamic Multiprocess Framework shares some similarities with Marsh and colleagues’ Attentional Allocation Policy view, according to which individuals distributed attentional resources between the ongoing and PM tasks based on their expectations about the context in which the PM cues occur (Cook et al., 2005; Marsh et al., 2005, 2006). In agreement with these views of strategic monitoring as a flexible mechanism, some studies found that participants tend to monitor only once they enter a context in which they expect to encounter a PM cue. This is accompanied with a reduced PM interference effect for blocks in which PM cue are not expected (Marsh et al., 2006; Cook et al., 2007). Interestingly, a study assessing retrieval experience based on self-report measures revealed that specifying the context wherein the PM cue would have been presented led to an increase in monitoring experiences. Furthermore, participants who received specific context information reported more frequent rehearsals of the PM task once such context occurred compared to when other contexts occurred (Meier et al., 2006). On the other hand, as also shown by Lourenço and Maylor (2014), when it was difficult to predict the occurrence of the PM cue (for example, during the switch between blocks in which the PM cue could occur and blocks in which the PM cue do not occur happened randomly and/or was not cued), a sort of monitoring process seemed to be engaged even if the intention was not relevant in that context (but see Smith and Loft, 2014). Furthermore, a series of studies exploring monitoring processes that are involved in time-based PM tasks—in which intentions must be completed at a specific time—consistently found that the frequency of clock checks increased as the PM target time was approaching (e.g., Ceci and Bronfenbrenner, 1985; Hicks et al., 2005; Mäntylä et al., 2009; Cona et al., 2012a,b). In time-based PM tasks, the occurrence of the PM cue is, indeed, intrinsically predictable, thus individuals can engage strategic monitoring in a flexible way, enhancing such process when it is greatly required.

Taking these studies together, it appears evident that the predictability of PM cues might play a crucial role in modulating the recruitment of strategic monitoring. However, the specific neural and cognitive mechanisms involved in predictable and unpredictable PM tasks remain less clear. Therefore, the aim of the present study is to explore the effect of PM cue predictability on the neurocognitive underpinnings of PM and, particularly, of strategic monitoring. Given the flexible nature of these processes, electrophysiological activity was measured along with behavioral performance in order to better characterize the temporal dynamic and the neural correlates of strategic monitoring processes.

The event-related potentials (ERPs) are frequently used to investigate the neural activity during PM tasks (e.g., West and Ross-Munroe, 2002; West and Krompinger, 2005; West, 2007; Zöllig et al., 2007; Cona et al., 2012a,b, 2014; Meier et al., 2014; see also a recent review by West, 2011). Great attention has been recently directed towards the ERP modulations associated with strategic monitoring processes (Knight et al., 2010; West et al., 2011; Cona et al., 2012a,b, 2014; Scolaro et al., 2014). Indeed, as compared with ongoing trials in blocks without the PM component, ongoing trials in PM blocks elicit sustained and generally more positive ERP modulations, starting around 200 ms after stimulus presentation and lasting for several hundred milliseconds. Such modulations are typically widespread over the scalp but particularly pronounced over the frontal regions (Cona et al., 2012a,b). These modulations are considered reflecting strategic monitoring processes such as the retrieval mode and the allocation of top-down attentional resources towards the environmental stimuli for target checking (West et al., 2011; Cona et al., 2012a,b; Czernochowski et al., 2012).

In the present study we explored the impact of predictable and unpredictable PM cues on the ERP modulations associated with strategic monitoring. We consider a PM cue as predictable if individuals have some degree of knowledge about its occurrence (i.e., the context in which the PM cue would appear), whereas we consider a PM cue as unpredictable if individuals do not have any clue about the PM cue occurrence. Based on previous findings (Marsh et al., 2006; Meier et al., 2006; Scullin et al., 2013; Lourenço and Maylor, 2014) we hypothesized that, with predictable PM cues, strategic monitoring-ERP modulations and the PM interference effect should be observed only in the context wherein individuals expect to encounter the PM cue, whereas these should not occur when the PM cue is not expected. By contrast, with unpredictable PM cues, strategic monitoring-ERP modulations and PM interference effect should occur during the whole experimental block since no information is given about when to expect the PM cue.

## Methods

### Participants

Forty-two students from the University of Padua participated in the experiment. All participants were right-handed, had no neurological pathologies, had normal or corrected-to-normal vision, and received course credits for their participation.

Each participant was randomly assigned to one of the two experimental conditions: Predictable or unpredictable condition. Data from one participant was discarded from the final analyses because of excessive artefacts in the EEG signal (i.e., more than 30% of trials rejected). The remaining 41 participants were distributed between the two experimental conditions as follows: 21 in the predictable condition and 20 in the unpredictable condition. Participants had a mean age of 24 years (SD = 3.78, range 19–42 years). Thirty participants were females and 11 were males. The study was approved by the ethical committee of the Psychology area of the University of Padua and was conducted according to the principles expressed in the Declaration of Helsinki. All the participants were informed about the general procedure of the experiment and signed a written consent form.

### Procedure

The participants of both the experimental PM conditions (predictable, unpredictable) were completed two blocks of trials. In the first block (i.e., baseline block), participants were only asked to perform the ongoing task. The baseline block included 200 ongoing task trials and was identical in both PM conditions.

**Figure 1:**
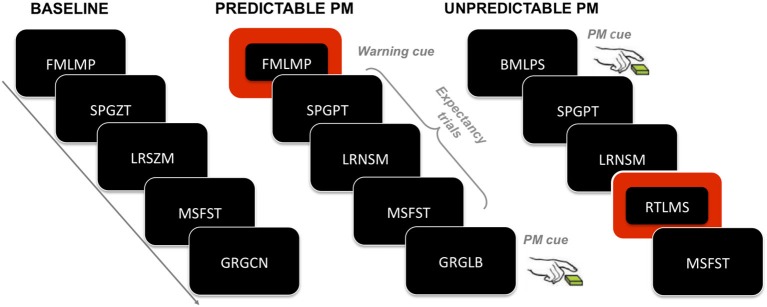
**Experimental paradigm**. The figure illustrates an example of sequence of trials for each task block. In the baseline block, participants have to merely execute the ongoing task (i.e., to decide whether the letters in the second and fourth positions are same or different). In the predictable and unpredictable conditions, participants have to execute not only the ongoing task but also the PM task, which consists in pressing a key whenever one of the letters in the first, third or fifth position of the letter string is a “B” letter (i.e., the PM cue). Participants assigned to the predictable condition are warned about the PM cue upcoming by means of a warning cue, which is represented by a switching of the color of the string background few trials before the occurrence of the PM cue; trials in between the warning cue and the PM cue are called expectancy trials. Participants assigned to the unpredictable condition are only told that in some trials the screen background would have changed from black to red, but that such change is not relevant to either the ongoing task or the PM task.

In the second block (i.e., PM block), participants were required to perform a PM task in addition to the ongoing task. The second block consisted of 400 ongoing trials and differed between the two conditions in the degree of information given about the PM cue occurrence. Therefore, the ongoing and the PM tasks were the same for both PM conditions. The only difference concerned the predictability of the PM cue occurrence (see Figure 1 for a schematic illustration of the experimental procedure).

### Ongoing Task

The ongoing task was similar to the task employed in our previous studies (e.g., Bisiacchi et al., 2011; Cona et al., 2012a,b) In each trial of the task, a string of five white letters was pseudo-randomly presented at the center of a black computer screen. However, unlike the stimuli used in our previous studies, the letters in the first, third and fifth positions always differed from each other. On the other hand, the letters in the second and fourth positions could be same or different. Participants were instructed to press the “N” key of the keyboard with their right index finger if the letters in the second and fourth positions were the same (e.g., SFMFD) and the “M” key with their right middle finger if the letters were different (e.g., LFDGT). Response keys were counterbalanced across participants. Each trial began with a blank screen with a pseudorandom duration ranging from 300 to 1200 ms. The five-letter string was then displayed either for 2000 ms or until the participants’ response (maximum response time = 2000 ms). A second blank screen followed the string presentation. The duration of this second blank screen was determined in such a way that overall duration of stimulus presentation plus the second inter-trial black interval was always 2000 ms.

### PM Task

The PM task consisted in pressing a response key (i.e., the “Z” key of the keyboard) with the left index whenever one of the letters in the first, third or fifth position of the letter string was a “B” letter (e.g., FLMLB). In such case, the participant had to perform the ongoing task first, and the PM task afterwards.

In the instructions given to the participants assigned to the predictable condition, it was specified that the PM cue would only appear in a given context. Participants were told that if, in a given trial, they saw a change in the screen background, i.e., changing from black to red (see Figure 1), a PM cue would appear during one of the succeeding trials. In this way, the trial having the red background acted as warning cue for signaling the upcoming occurrence of the PM cue. After the warning cue, the background color switched back to black. The PM cue could appear after 3, 4 or 5 trials after the warning cue. There were a total of 15 background color changes (i.e., warning cues) across the experiment and, consequently, a total of 15 PM cues.

Participants assigned to the unpredictable condition were told that the PM cue could appear anytime throughout the whole experiment. Participants were also informed that in some trials the screen background would have changed from black to red, but that such change was not relevant to either the ongoing task or the PM task. The PM cue never appeared during the red background trials.

### EEG Pre-Processing

EEG signal was recorded as in the studies by Cona et al. (2012a,b). The EEG signal was acquired with the System Plus equipment (Micromed, Mogliano Veneto, Italy) using an array of 32 Ag/Ag Cl scalp electrodes mounted on an elastic cap (ElectroCap International, Inc.). Electrodes were positioned according to the 10–20 International System. The scalp montage included the following electrode positions: Fp1, Fpz, Fp2, AFz, F7, F3, Fz, F4, F8, Fc3, Fcz, Fc4, T3, C3, Cz, C4, FT7, FT8, T3, T8, T5, Cp3, Cpz, Cp4, P3, Pz, P4, T6, Tp7, Tp8, O1, O2. Four additional electrodes were used to record signal from left and right mastoid, and for recording eye movements. Eye movements were monitored by two electrodes, one electrode placed above the right eye, and one placed on the external *canthi* of the left eye. The EOG (electrooculogram) was recorded with a bipolar montage. All scalp electrodes were referenced to the left mastoid and re-referenced offline to the average of the left and right mastoids. The ground electrode was placed in AFz. Data were digitized at a sampling rate of 512 Hz. Electrode impedance was always kept below 5 kΩ throughout the recording session. Data processing was performed with EEGLAB 12.0.2 (Delorme and Makeig, 2004), running under the Matlab environment (Version 7.4.0, MathWorks, Natick, MA, USA). Continuous EEG data was filtered between 0.1 Hz and 30 Hz and resampled at 256 Hz. Then, it was segmented into epochs starting −200 ms before the onset of the stimulus and ending 1200 ms after the onset of the stimulus. Epochs were locked to the presentation of the ongoing stimuli (i.e., letter strings). Artefact correction was done on these epochs by using the Independent Component Analysis (ICA) toolbox in EEGLAB. Only epochs with correct responses (to the ongoing and PM tasks) were selected. Epoch rejection was performed with a threshold of ±100 μV. The final epochs were averaged offline separately for each experimental condition. ERP measures were calculated using the *erpR* package, version 0.2.0, (Arcara and Petrova, 2014) running under R 3.1.0 (R Core Team, 2014). All ANOVA analyses were performed using the *ez* R package (Lawrence, 2013).

### Statistical Analysis

According to the experimental design, ERP and behavioral data were averaged separately for each type of trial. The *warning cues* referred to the trials having a red screen background. The *expectancy* trials included, in the predictable condition, the trials occurring between the warning cue and the PM cue, whereas, in the unpredictable condition, the four trials following the warning cue. It is important to note that, for the unpredictable condition, the *warning cue* and the *expectancy* trials included trials that were perceptually identical to those of predictable condition. However, given the different instructions, these trials were uninformative about the appearance of PM cue. Therefore, the terms “*warning cue*” and “*expectancy trials*” were given based on the meaning of such types of trials in relation to predictable condition. The *PM cue* referred to the trials in which the participants were required to perform the PM task. The *ongoing trials* in the baseline block included all trials in the first block. Finally, the *ongoing trials* in the PM block included all ongoing trials in the PM block, except the previous described types of trial. The ERP analyses were based on an average of: 185.9 (SD = 22.75) ongoing trials in the baseline block, 288.8 (SD = 43.47) ongoing trials in the PM block, 56.4 (SD = 8.46) expectancy trials, 13.0 (SD = 2.06) warning cue trials, and 12.8 (SD = 2.70) PM cue trials for the group in the predictable condition; and of 189.8 (SD = 8.47) ongoing trials in the baseline block, 243.1 (SD = 7.77) ongoing trials in the PM block, 57.9 (SD = 1.68) expectancy trials, 13.5 (SD = 1.27) warning cue trials, and 13.2 (SD = 1.28) PM cue trials for the group in the unpredictable condition.

### Behavioral Data Analysis

The data in the ongoing task were analyzed by means of two separate ANOVAs, which both included mean RTs and mean accuracy (i.e., proportion of correct responses) in the ongoing trials as dependent variables. The first analysis consisted of a mixed 2 × 2 ANOVA, with Predictability Condition (predictable, unpredictable) as the between-subject factor and Block (baseline block, PM block) as the within-subject factor. The second analysis took into account all trial types in the PM block. This mixed 2 × 4 ANOVA included Predictability Condition (predictable, unpredictable) as the between-subject factor and Trial Type (ongoing trial, warning cue, expectancy trial, and PM cue) as the within-subject factor. In all the analyses, Bonferroni correction was applied for multiple comparisons.

A further analysis was run to investigate the proportion of correct responses to the PM task as a function of the predictability condition: A *t*-test was carried out to contrast the performance on the PM task between the two experimental predictability conditions.

### ERP Data Analysis

The analyzed ERP waveforms were time-locked to the presentation of the ongoing stimuli. In all the analyses, the dependent variable was the mean voltage amplitude in selected time windows. Since the ERPs elicited by the experimental conditions differed remarkably in topography and morphology, ERP data were examined in four different clusters of analysis, defined according to the experimental design and hypotheses of the study. In each cluster of analysis, distinct time windows and electrodes were selected on the basis of both visual inspection of the effects and previous literature.

The first cluster of analysis investigated the differences on the ERPs elicited by ongoing trials between the baseline block and the PM block, comparing them as a function of the cue predictability condition (predictable, unpredictable). The ERP modulations associated with the addition of the PM task revealed slow wave activity with bilateral fronto-parietal distribution that closely resembled the ERP activity observed in studies using similar experimental tasks (Cona et al., 2012a,b). Following past literature (e.g., Cona et al., 2012a), three separate mixed ANOVAs were carried out for the following three time windows: 200–400 ms, 400–700 ms, 700–1000 ms. In each of the three ANOVAs, three factors were considered: Predictability condition (predictable, unpredictable), Block (baseline block, PM block), and Electrode as a within-subject factor (F7, F8, P3, P4).

The second cluster of analysis investigated the differences between ongoing trials and the expectancy trials as a function of the predictability condition. As compared with the ERPs in ongoing trials of PM block, the ERPs elicited by the expectancy trials in the predictable condition were indeed characterized by a sustained increased positive deflection over antero-lateral electrodes, coupled with a reduced transient positive component over parietal electrodes. Two separate ANOVAs were carried out for the following time windows: 300–500 ms and 700–1000 ms. Each ANOVA included: Predictability condition (predictable, unpredictable), Trial type as a within-subject factor (ongoing trials, expectancy trials), and Electrode (F7, F8, P3, P4).

The third cluster of analysis investigated the differences in the ERPs elicited by the warning cues in relation to predictability condition. The waveforms of ERPs elicited by the warning cues were characterized by a pronounced P3a deflection over fronto-central, central and centro-parietal electrodes. Two separate ANOVAs were carried out for the following time windows: 300–400 ms and 400–500 ms. The two time windows investigated the predictability effects on the peak and on the descending arm of the P3a component, respectively. Each of the two ANOVAs included Predictability condition (predictable, unpredictable), Trial type (ongoing trials, warning cues), and Electrode (FC3, FC4, CP3, CP4) as factors.

The fourth cluster investigated the ERP differences in the PM trials as a function of the predictability condition. As compared with the ongoing trials, the ERP waveforms of PM trials (in predictable condition) in the 300–600 ms time window were characterized by a single positive deflection, which was particularly expressed in fronto-central electrodes and in parietal electrodes. To investigate this effect, a mixed ANOVA was carried out in the 300–600 ms time window. This ANOVA included Predictability condition (predictable, unpredictable), Trial type (ongoing trials, PM cues), and Electrode (FC3, FC4, P3, P4) as factors.

In the ERP analyses, *post hoc* comparisons were carried out by means of *t*-tests with False Discovery Rate (FDR) correction method.

In all behavioral and ERP ANOVAs analyses, sphericity assumption was checked by means of Mauchly test. When necessary, Greenhouse-Geisser correction was applied and corrected *p*-*values* are reported. Effect size was calculated by means of generalized eta squared (Bakeman, 2005).

### Behavioral Results

Mean values of RTs and proportion of correct responses in the ongoing task are reported separately for each experimental condition, in Table 1.

**Table 1:** **Behavioral measures in the ongoing task**.

	Ongoing trials in baseline block	Ongoing trials in PM block	Warning cues	Expectancy trials	Ongoing trials containing a PM cue
*Reactions Times (in ms) in the Ongoing task*
**Predictable condition**	749 (102)	790 (115)	1024 (181)	915 (145)	1194 (207)
**Unpredictable condition**	747 (123)	889 (179)	1015 (143)	883 (178)	1088 (185)
*Mean Accuracy in the Ongoing task*
**Predictable condition**	0.97 (0.02)	0.96 (0.05)	0.89 (0.06)	0.97 (0.04)	0.93 (0.07)
**Unpredictable condition**	0.97 (0.02)	0.97 (0.01)	0.92 (0.09)	0.98 (0.02)	0.96 (0.04)

*Mean values (SD) of RTs in ms and accuracy, expressed as proportion of correct responses, across all experimental conditions*.

The analysis of ongoing RTs in the baseline and PM blocks for the two predictability conditions revealed a significant main effect of Block (*F*_(1,39)_ = 46.98, *p* < 0.001, $\eta_{G}^{2}$ = 0.10), as well as a Block × Predictability condition interaction (*F*_(1,39)_ = 14.72, *p* = 0.004, $\eta_{G}^{2}$ = 0.03). *Post hoc* analysis investigating such interaction showed that the ongoing task RTs were slower in the PM block than in the baseline block only for the unpredictable condition (*p* < 0.001), but not for the predictable condition (*p* > 0.05). The RTs in PM block for unpredictable condition tend to be slower than RTs in PM block of predictable condition, since this difference approached significance (*p* = 0.06, uncorrected *p* = 0.04).

In the analysis of RTs for each Trial type within PM block, two effects were significant: the main effect of Trial type (*F*_(3,117)_ = 118.43, *p* < 0.001, $\eta_{G}^{2}$ = 0.33) and the Predictability condition × Trial type interaction (*F*_(3,117)_ = 11.75, *p* < 0.001, $\eta_{G}^{2}$ = 0.05). *Post hoc* analysis showed that, in the predictable condition, all trials types significantly differed from each other. The RTs were fastest in the ongoing trials, followed by the RTs in the expectancy trials, then by RTs for the warning cues, and finally by the PM cues, which elicited the slowest RTs (all *ps* < 0.001). Similar to the predictable condition group, unpredictable condition group exhibited slower RTs in warning cues than in both the expectancy and ongoing trials, and slowest RTs the PM trials (all *ps* < 0.05). Nevertheless, it’s noteworthy that in the unpredictable condition group, the ongoing trials did not significantly differed from expectancy trials (*p* = 0.49).

The comparison of accuracy in the ongoing task between the baseline block and the PM block for the two predictability conditions did not show any significant effect. Instead, the analysis of accuracy in the ongoing task as a function of the distinct Trial type showed a significant effect of Trial type (*F*_(3,117)_ = 20.16, *p* < 0.001, $\eta_{G}^{2}$ = 0.20). Accuracy was lower for the warning cue and for the PM cue trials compared to both the ongoing trials and the expectancy trials (*ps* < 0.05). The lowest accuracy was found for the warning cue trials, which also differed significantly from the PM cue trials (*p* < 0.05).

Concerning the PM performance, the mean proportion of accuracy in the PM task was 0.87 (SD = 0.13) for the predictable condition and 0.80 (SD = 0.14) for the unpredictable condition. This difference was not significant (*t*_(39)_ = 1.43, *p* > 0.05).

### ERP Results

#### Ongoing Trials in Baseline Block vs. PM Block

##### 200–400 ms

The analysis of the ERPs in the 200–400 ms time windows, locked to the ongoing trials in the baseline block and in the PM block, for both the predictable and unpredictable conditions, showed a significant main effect of Block (*F*_(1,39)_ = 11.50, *p* = 0.002, $\eta_{G}^{2}$ = 0.01), a main effect of Electrode (*F*_(3,117)_ = 3.50, *p* = 0.02, $\eta_{G}^{2}$ = 0.03), and a Block × Electrode interaction (*F*_(3,117)_ = 4.28, *p* = 0.006, $\eta_{G}^{2}$ = 0.003). *Post hoc* comparisons showed that in the F7 and F8 electrodes, the mean amplitude of the ERPs was higher in the PM block than in the baseline block, regardless of the predictability condition (*ps* < 0.01).

##### 400–700 ms

**Figure 2:**
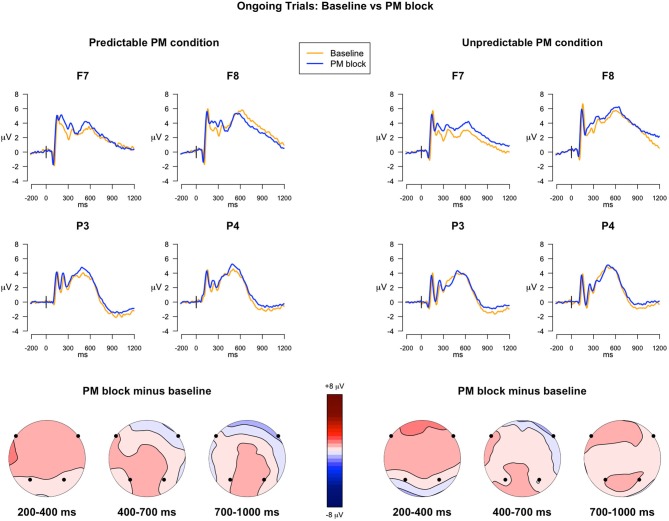
**ERPs in the ongoing trials: Baseline *vs.* PM block**. The upper panel shows the grand mean ERPs at frontal (F7, F8) and parietal (P3, P4) recording sites, averaged across participants. The ERPs are depicted separately for predictable and unpredictable condition, with the waveforms for the baseline block (*yellow line*) and PM block (*blue line*) superimposed. The lower panel shows the scalp topography of the difference between amplitude means in the PM block and the baseline block, separately for the predictable and unpredictable condition, obtained by interpolating the values from all the 32 recording sites in the 200–400 ms, 400–700 ms and 700 ms time windows. The dots highlight the electrodes taken into account in the analyses.

In the second time window, the ANOVA showed the following significant effects: Electrode (*F*_(3,117)_ = 5.78, *p* = 0.009, $\eta_{G}^{2}$ = 0.06), Block × Electrode (*F*_(3,117)_ = 3.28, *p* = 0.04, $\eta_{G}^{2}$ = 0.002), and Predictability condition × Block × Electrode (*F*_(3,117)_ = 3.85, *p* = 0.02, $\eta_{G}^{2}$ = 0.003). Notably, *post hoc* comparisons of the interaction revealed that, in F7, the ERP amplitude was significantly more positive in the PM block than in the baseline block, only for the unpredictable condition (*p* = 0.01), whereas no significant differences were observed in the predictable condition (Figure 2).

##### 700–1000 ms

In the time window encompassing late components, the effect of Electrode was significant (*F*_(3,117)_ = 73.85, *p* < 0.001, $\eta_{G}^{2}$ = 0.37), as well as the Block × Electrode interaction (*F*_(3,117)_ = 4.77, *p* = 0.01, $\eta_{G}^{2}$ = 0.006). The *post hoc* analysis investigating the interaction did not show any significant difference, nevertheless uncorrected comparisons showed a significant difference in F7 and P4, with higher mean amplitude in PM block than in the baseline block, regardless of the predictability condition.

#### Expectancy Trials vs. Ongoing Trials in PM Block

##### 300–500 ms

**Figure 3:**
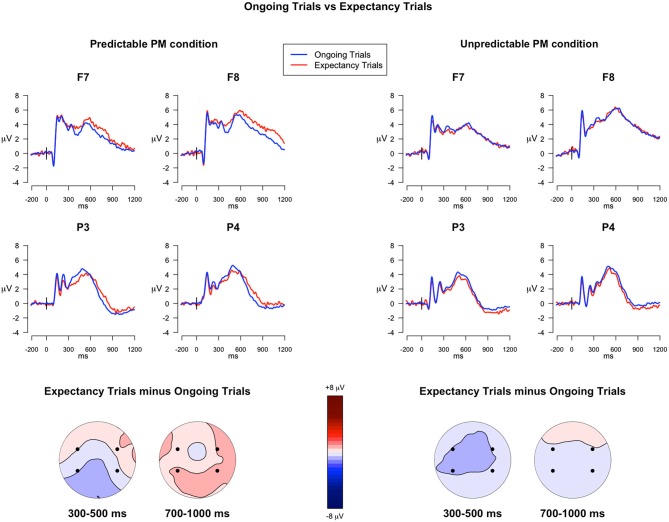
**ERPs in the ongoing trials *vs.* in the expectancy trials**. The upper panel shows the grand mean ERPs at frontal (F7, F8) and parietal (P3, P4) recording sites, averaged across participants. The ERPs are depicted separately for predictable and unpredictable conditions, with the waveforms for the ongoing PM trials (*blue line*) and expectancy trials (*red line*) superimposed. The lower panel shows the scalp topography of the difference between amplitude means for the expectancy trials and the ongoing trials, separately for predictable and unpredictable conditions group, obtained by interpolating values from all the 32 recording sites in the 300–500 ms and 700–1000 ms time windows. The dots highlight the electrodes taken into account in the analyses.

The analysis investigating the ERP differences between the expectancy trials and the ongoing trials, in predictable and unpredictable conditions, revealed two significant interactions: Trial type × Electrode (*F*_(3,117)_ = 8.23, *p* < 0.001, $\eta_{G}^{2}$ = 0.003) and Trial type × Predictability condition × Electrode (*F*_(3,117)_ = 3.99, *p* = 0.009, $\eta_{G}^{2}$ = 0.001). Since the *post hoc* comparisons investigating the triple interaction showed differences approaching significance (*p* < 0.07), the uncorrected *p*-values were taken into account. The comparisons revealed that, only for the predictable condition, the ERP components in expectancy trials were significantly more positive in F8 and less positive in P3 compared to the ERPs in ongoing trials (*ps* < 0.05), whereas no differences were observed in the unpredictable condition (See Figure 3). All the other within-group and between-group comparisons were not significant.

##### 700–1000 ms

In the analysis of the late time window, the main effect of Trial Type was significant (*F*_(1,39)_ = 7.52, *p* = 0.009, $\eta_{G}^{2}$ = 0.007), as well as the main effect of Electrode (*F*_(3,117)_ = 64.95, *p* < 0.001, $\eta_{G}^{2}$ = 0.37) and the Trial Type × Predictability condition interaction (*F*_(1,39)_ = 17.19, *p* < 0.001, $\eta_{G}^{2}$ = 0.01). The *post hoc* comparisons investigating the Trial type × Predictability condition interaction showed that, in all the selected electrodes, the expectancy trials had higher mean amplitudes relative to the ongoing trials (Figure 3), but only for the predictable condition (*p* < 0.001). All the other within-group and between-group comparisons were not significant.

#### Warning Cues vs. Ongoing Trials in PM Block

##### 300–400 ms

**Figure 4:**
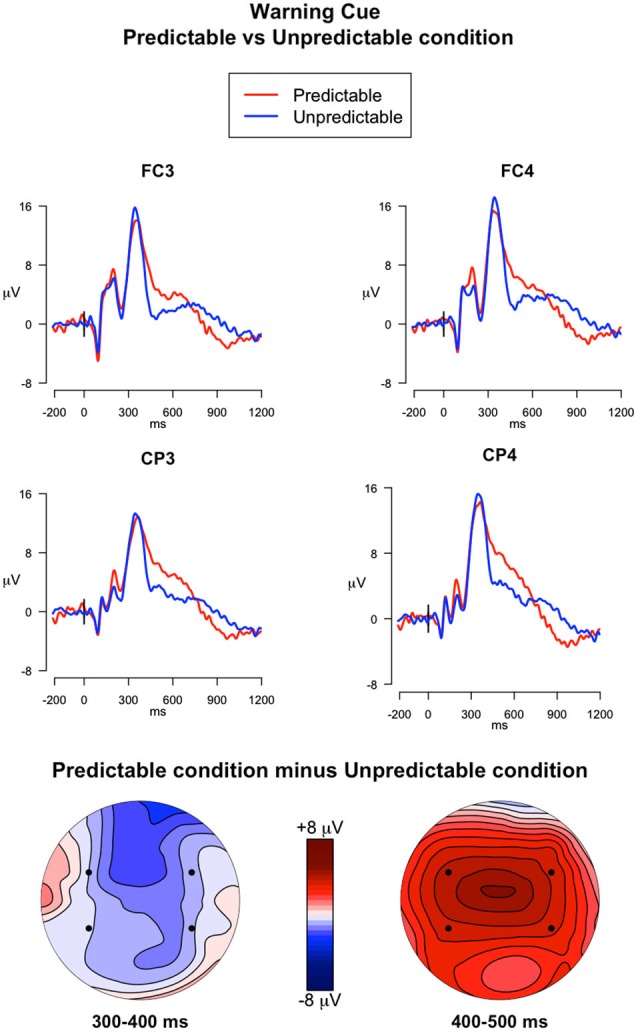
**ERPs elicited by the warning cues**. The upper panel shows the grand mean ERPs at fronto-central (FC3, FC4) and centro-parietal (CP3, CP4) recording sites averaged across participants, with the waveforms in the predictable condition (*red line*) and unpredictable condition (*blue line*) superimposed. The lower panel shows the scalp topography of the difference between amplitude means for the predictable vs. unpredictable warning cues, obtained by interpolating values from all the 32 recording sites in the 300–400 ms and 400–500 ms time windows. The dots highlight the electrodes taken into account in the analyses.

The analysis investigating the effect of PM cue predictability on the ERPs elicited by the warning cues captured the P3a peak in the 300–400 ms time window (Figure 4). In this analysis the following effects were significant: a main effect of Electrode (*F*_(3,117)_ = 6.24, *p* < 0.001, $\eta_{G}^{2}$ = 0.02), a main effect of Trial Type (*F*_(1,39)_ = 203.71, *p* < 0.001, $\eta_{G}^{2}$ = 0.56), and a Trial Type × Electrode interaction (*F*_(3,117)_ = 10.65, *p* < 0.001, $\eta_{G}^{2}$ = 0.008). The main effect of Trial Type indicates higher mean amplitudes in the warning cues compared to the ongoing trials. *Post hoc* comparisons exploring the interaction revealed that ERP amplitudes were higher in the warning cues than in ongoing trials in all the electrodes selected (all *ps* < 0.001). Moreover, in the ongoing trials, the ERP amplitudes were higher in FC3 than in FC4 and CP3 (*ps* < 0.001), whereas in the warning cues, the amplitudes were higher in FC4 than in FC3 and CP3 (*ps* < 0.001).

##### 400–500 ms

The analysis of the descending arm of the P3a (Figure 4) showed a significant main effect of Electrode (*F*_(3,117)_ = 12.39, *p* < 0.001, $\eta_{G}^{2}$ = 0.03), a significant main effect of Trial type (*F*_(1,39)_ = 15.31, *p* < 0.001, $\eta_{G}^{2}$ = 0.08), and a significant Trial type × Predictability condition interaction (*F*_(1,39)_ = 8.09, *p* = 0.007, $\eta_{G}^{2}$ = 0.05). *Post hoc* comparisons investigating the interaction showed that the ERPs elicited by the Warning cues had higher mean amplitudes in the predictable condition compared to the unpredictable condition in all selected electrodes (*p* < 0.05).

#### PM Cues vs. Ongoing Trials in PM Block

##### 300–600 ms

**Figure 5:**
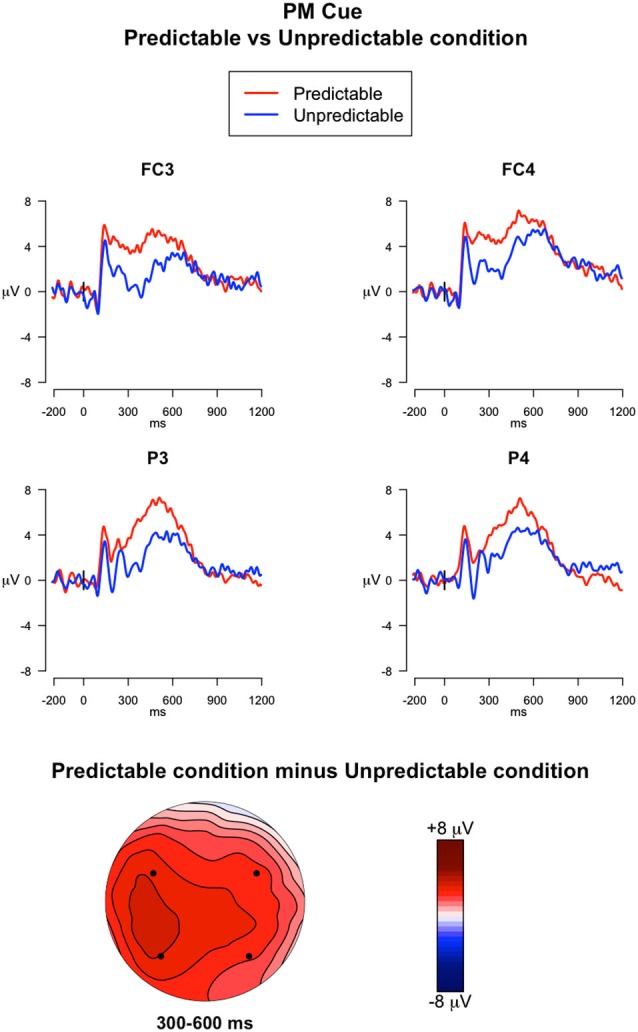
**ERPs elicited by the PM cues**. The upper panel shows the grand mean ERPs at fronto-central (FC3, FC4) and parietal (P3, P4) recording sites averaged across participants, with the waveforms in the predictable condition (*red line*) and unpredictable condition (*blue line*) superimposed. The lower panel shows the scalp topography of the difference between amplitude means for the predictable vs. unpredictable PM cues, obtained by interpolating values from all the 32 recording sites in the 300–600 ms time window. The dots highlight the electrodes taken into account in the analysis.

The ANOVA run on mean amplitude in this time window revealed a significant main effect of Electrode (*F*_(3,117)_ = 3.23, *p* = 0.02, $\eta_{G}^{2}$ = 0.01) and a Trial type × Predictability condition interaction (*F*_(1,39)_ = 13.66, *p* < 0.001, $\eta_{G}^{2}$ = 0.01). *Post hoc* comparisons of the Trial type × Predictability condition interaction showed that the PM cues had higher amplitudes relative to the ongoing trials only for the predictable condition (*p* < 0.05). Importantly, the amplitude measured during the PM cues was significantly higher in predictable condition than in the unpredictable condition (*p* < 0.05) (Figure 5).

## Discussion

The present study was designed to investigate the effect of PM cue predictability on neurocognitive processes underlying prospective remembering. The results revealed modulations of behavioral and ERP measures as a function of PM cue predictability that seem to support the Dynamic Multiprocess Framework according to which strategic monitoring is a flexible mechanism recruited especially when the PM cue is expected (Scullin et al., 2013).

The allocation of strategic monitoring resources to the PM task is typically reflected in the PM interference effect, which is the decline in ongoing task performance when a PM task is added to the ongoing activity relative to a baseline block without the PM instructions (Marsh et al., 2003). In our study, only the participants of the unpredictable condition showed the PM interference effect on the ongoing task RTs, whereas the participants of the predictable condition did not show slower ongoing task RTs in the PM block compared to the baseline block. In the predictable condition participants were informed that the PM cue would not occur in those ongoing trials, so they did not expect to encounter it and, consequently, they did not devote resources for strategic monitoring. By contrast, the uncertainty about the occurrence of the PM cue in the unpredictable condition led to an overall higher need for strategic monitoring given that attentional resources were distributed evenly throughout the whole block. Further evidence of this explanation is provided by the comparison between the RTs in the expectancy trials (i.e., ongoing trials occurring right after the warning cue) and the RTs in the ongoing trials. This comparison revealed a slowing down of RTs only in the expectancy trials of the predictable condition. In the predictable condition participants knew that the PM cues would occur in those trials, so they started monitoring for the PM cue occurrence. On the other hand, in the unpredictable condition, participants did not think that it was more likely to encounter a PM cue in the expectancy trials than in the ongoing trials, therefore they did not increase the amount of resources devoted for strategic monitoring in expectancy trials. The pattern of ERP results tells a similar story while offering a more detailed account of the neurocognitive underpinnings of PM and strategic monitoring. We found that the ERP modulations that were typically associated with strategic monitoring were differently expressed in ongoing trials depending on PM cue predictability. In general, as compared with trials of the baseline block, ongoing trials of the PM block elicited sustained positive modulations that emerged 200 ms after stimulus presentation, persisted approximately until 1000 ms and were distributed mainly over the anterior and lateral frontal regions (Figure 2), corroborating previous ERP studies on strategic monitoing (Cona et al., 2012a,b, 2014, 2015a; Czernochowski et al., 2012; Scolaro et al., 2014). Nevertheless, at a closer inspection, the earlier (200–400 ms) and the later parts (700–1000) of these modulations tended to emerge in both predictable and unpredictable conditions, whereas the central part was significant (400–700 ms) only in the unpredictable condition. This pattern of modulations suggests that multiple cognitive processes would contribute to originate such frontal positivity, being differently modulated by PM cue predictability. Specifically, retrieval mode, in which the intention is actively maintained in mind, has been associated with long-lasting slow wave activity. Such activity is not strictly related to the stimulus onset but also observed in the response-to-stimulus interval when no stimulus is present (West et al., 2011). In our study, the earlier and the later time windows might highlight such a slow wave activity related to retrieval mode, suggesting that this process is engaged in both predictable and unpredictable conditions. The overlapping modulation that emerged between 400–700 ms only in the unpredictable condition, with the ERPs over frontal regions being more positive in the PM block than in the baseline block, might instead represent the ERP correlate of target checking, which seemed to be involved only in the ongoing trials of the unpredictable condition. This result is consistent with previous studies that reported modulations associated with target checking in similar time windows (e.g., Cona et al., 2012a). Furthermore, the distribution of the slow wave activity over lateral frontal regions would be in line with the gateway hypothesis, which assumed that lateral regions of prefrontal cortex play a crucial role in the maintenance of intention in mind (Burgess et al., 2007, 2011).

We hypothesized that, in predictable condition, strategic monitoring—and more specifically target checking—was recruited in the expectancy trials in order to monitor for the occurrence of the PM cue. The comparison of the ERPs elicited by expectancy trials with the ERPs elicited by ongoing trials seems to confirm our hypothesis. Indeed, in the predictable condition, expectancy trials were characterized by an increased sustained positivity compared to the ongoing trials (Figure 3). Such positivity started around 300 ms post-stimulus and lasted until 1000 ms, and was expressed over lateral frontal regions, especially the right ones and, in a later time window (700–1000 ms), also over parietal regions. As in the behavioral results, we did not find difference in the ERPs between expectancy trials and ongoing trials for participants in the unpredictable condition. This finding could be explained by the fact that they were continuously engaged in target checking, thus leading to no differences between ongoing and expectancy trials.

It is noteworthy that, for the predictable condition, there was a transient modulation between 300–500 ms post-stimulus over left parietal regions. This is likely to represent a reduction in the P3b for the expectancy trials relative to the ongoing trials. The P3b is considered to reflect stimulus identification and it is sensitive to the degree of attentional resources allocated to the stimulus (Polich, 2007). A possible explanation of the reduction of P3b might be that, in the expectancy trials of predictable condition, the attention was partially directed away from the stimulus attributes that were relevant to ongoing task in favor of processing other stimulus attributes, important for detecting the PM cue. In fact, since the PM cue was nonfocal, the process required for the ongoing task did not direct attention towards processing the features of the PM cue. This explanation is in agreement with results from dual-task studies, which revealed that increasing the difficulty of one of the tasks led to fewer resources for the other task, and this would be reflected in decrease of P3b amplitude (e.g., Kok, 2001; Watter et al., 2001). In such a way, the reduction of the P3b might reflect the disengagement of attention from ongoing stimulus-dependent processes in service to stimulus-independent processes, such as activation of PM intention (i.e., “the gateway hypothesis”; Burgess et al., 2007). Although the spatial resolution of the ERP technique is relatively low, the present result seems to also be in line with the Attention to Delayed Intention (AtoDI) model, according to which parietal regions are involved in the allocation of attention both externally—to detect the PM stimuli—and internally—to activate the intention-related contents (Cona et al., 2015b).

Insights about the processes involved in predictable and unpredictable PM tasks were also provided by the ERPs elicited by the warning cue and the PM cue. As compared with the ERPs in the ongoing trials, the ERPs elicited by the warning cues were characterized by a pronounced frontal and central positive component that occurred in the time window between 200–400 ms post-stimulus for the unpredictable condition and within the 200–500 ms time window for the predictable condition (Figure 4). The modulation reflects the P3a, thus suggesting a bottom-up capture of attention by the warning cue, likely because it was a salient stimulus (Schröger and Wolff, 1998; Friedman et al., 2001; Cona et al., 2013a). The distracting nature of the warning cue was testified by the slower RTs for these trials respect to the ongoing trials. Moreover, the P3a showed a slower return to the isoelectric line in the predictable condition relative to the unpredictable condition. For participants in the unpredictable condition, the warning cue was irrelevant given that it did not carry with itself any meaning or information about either the ongoing or PM task. Therefore, in the unpredictable condition, their attention was promptly disengaged away from the warning cue to be refocused towards the upcoming ongoing stimulus, as suggested by the reorienting negativity (RON) occurring after the P3a (Schröger and Wolff, 1998; Munka and Berti, 2006; Berti, 2008; Cona et al., 2013b). Conversely, in the predictable condition, another positive component overlaps the P3a, thus originating a slower return of the P3a to the isoelectric line. This component might reflect the retrieval of intention from memory. In the predictable condition the role of the warning cue was to inform participants that the PM cue would occur within the next few trials. Therefore, in this condition, the warning cue seems to automatically activate the PM intention stored in mind. This idea is supported by the timing (400–500 ms time window) and topography of the modulation, which are typical of the FN400, an ERP component related to automatic memory processes (Curran, 2000). The present result corroborates the most recent studies and theories stating that salient stimuli tend to trigger a bottom-up capture of attention directed to intention, which can be spontaneously retrieved from memory (the “Dynamic Multiprocess Framework”, Scullin et al., 2013; the Attention to Delayed Intention (AtoDI) model, Cona et al., 2015b).

Finally, the predictability of the PM cue affected the ERPs elicited by the PM cue itself. Increased sustained positivity was observed for predictable PM cues relative to both unpredictable PM cues and ongoing trials. Such positivity occurred in the time window approximately between 300–600 ms and was widespread over the scalp, being expressed from frontocentral to parietal regions of the scalp (Figure 5). This modulation seems to reflect the increased amount of top-down attentional resources allocated to process the PM cue in the predictable cue condition (Cona et al., 2015b). In fact, since participants expected to encounter the PM cue, they were prepared to detect it and to retrieve the associated intention by recruiting preparatory top-down processing resources. This result is consistent with a recent study, showing an increase in the ERP components, in a similar time window, during habitual PM tasks (Meier et al., 2014). In line with our explanation, such enhanced amplitude was interpreted as reflecting reallocation of processing resources, or a facilitation of intention retrieval, or a combination of both these factors.

In conclusion, the predictability of the PM cue is revealed to be a crucial factor in modulating the engagement of strategic monitoring given that it can influence the attentional allocation policy (Marsh et al., 2006; Meier et al., 2006). Continuous monitoring for PM cues would be time-consuming and wasteful of attentional resources that could otherwise be allocated to accomplish ongoing activities. Therefore, when the occurrence of the PM cue can be predicted, individuals seem to recruit strategic monitoring only when they enter in a context wherein the PM cue is expected, whereas they flexibly disengage strategic monitoring when the PM cue is not soon encountered (e.g., Meier et al., 2006; Scullin et al., 2013; Lourenço and Maylor, 2014). This result is consonant with the studies using time-based paradigms (e.g., Ceci and Bronfenbrenner, 1985; Hicks et al., 2005; Mäntylä et al., 2009; Cona et al., 2012a,b), and with the study by Meier et al. (2006), which, though using a different approach, has shown that entering in a context wherein the occurrence of a PM cue was expected gave rise to an increase in self-reported monitoring experiences.

Conversely, when individuals have no clues about the PM cue occurrence, they cannot decide how to clearly distribute their cognitive resources between the ongoing and the PM task in advance. Hence, they tend to engage in strategic monitoring in a more continuous manner. It is important to note, however, that our experimental situation did not last very long, so it was advantageous to spread attentional resources throughout all the task session. A parallel example in everyday life might be forming the intention to refuel the car while you are driving in an unknown street. In this situation, you might be continuously engaged in monitoring the street for the occurrence of a gas station. Nevertheless, when the lapse of time between the formation and the realization of an intention is long, even though the occurrence of the PM cue was relatively unpredictable, you probably would not recruit strategic monitoring constantly, since this would be disadvantageous. This hypothesis is also driven by a study showing that, when PM cue occurrence could not be predicted, the number of monitoring experiences linearly decreased as the delay of retrieval of intentions increased (Meier et al., 2006). Therefore, a question for future studies should be how the interplay between the “cue predictability” and “delay of intention” factors can modulate the neural underpinnings of PM processes, and in particular, strategic monitoring.

## Conflict of Interest Statement

The authors declare that the research was conducted in the absence of any commercial or financial relationships that could be construed as a potential conflict of interest.
